# Visual objects approaching the body modulate subsequent somatosensory processing at 4 months of age

**DOI:** 10.1038/s41598-023-45897-4

**Published:** 2023-11-21

**Authors:** Giulia Orioli, Irene Parisi, José L. van Velzen, Andrew J. Bremner

**Affiliations:** 1 Centre for Developmental Science, School of Psychology, University of Birmingham, Birmingham, UK; 2 Department of Psychology, Goldsmiths, University of London, London, UK; 3 Present Address: Department of Psychology, Sapienza, University of Rome, Rome, Italy

**Keywords:** Perception, Sensory processing, Somatosensory system, Sensorimotor processing, Human behaviour

## Abstract

We asked whether, in the first year of life, the infant brain can support the dynamic crossmodal interactions between vision and somatosensation that are required to represent peripersonal space. Infants aged 4 (n = 20, 9 female) and 8 (n = 20, 10 female) months were presented with a visual object that moved towards their body or receded away from it. This was presented in the bottom half of the screen and not fixated upon by the infants, who were instead focusing on an attention getter at the top of the screen. The visual moving object then disappeared and was followed by a vibrotactile stimulus occurring later in time and in a different location in space (on their hands). The 4-month-olds’ somatosensory evoked potentials (SEPs) were enhanced when tactile stimuli were preceded by unattended approaching visual motion, demonstrating that the dynamic visual-somatosensory cortical interactions underpinning representations of the body and peripersonal space begin early in the first year of life. Within the 8-month-olds’ sample, SEPs were increasingly enhanced by (unexpected) tactile stimuli following receding visual motion as age in days increased, demonstrating changes in the neural underpinnings of the representations of peripersonal space across the first year of life.

## Introduction

Acting in the environment and perceiving one’s place in it requires the ability to represent the dynamic relations between sensory events originating in external space (often audiovisual features of objects or people) and sensory events on the body (often somatosensory stimuli)^1–3^. The last 20 years have seen significant advances in our understanding of this ability to represent events in peripersonal space^4,5^. Given the dynamic nature of peripersonal spatial interactions—where events in external (visual and auditory) space influence subsequent somatosensory events and vice versa—more recent investigations have considered the role of sensory predictions^6–8^ in underpinning human adults’ representations of peripersonal space^1,9–11^. Whereas a handful of studies have investigated the development of multisensory space in human infancy^12,13^, no research has yet examined the development of an ability to perceive the dynamic multisensory interactions across spatial and temporal gaps that underpin the perception of sensory events characterising the interaction of the body with its environment. Here we begin to address this by reporting the findings of an investigation into the influences, in 4- and 8-month-old human infants, of visual motion in extrapersonal space (towards or away from the body) on the processing of somatosensory stimulation occurring on the body after a temporal interval.

Over the years there have been a number of attempts to trace the origins of human infants’ ability to perceive sensory events in relation to the self^14–20^. Orioli et al.^19,20^ have recently demonstrated that even newborns can differentiate visual events based on their motion direction relative to the body, showing a visual preference for objects moving towards them versus away from them. Other studies have established that from early in the first year infants are sensitive to temporal and spatial multisensory contingencies between visual, auditory and tactile stimuli that are likely to play a fundamental role in peripersonal space representations^12,21–26^. What remains unclear is the extent to which early sensitivity to such multisensory contingencies can support infants’ representations of dynamic events in peripersonal space. Such a representational ability requires the encoding of visual-tactile correspondences across spatiotemporal gaps (e.g., when seeing an object moving in extrapersonal space that subsequently touches the body)^9,10^.

Recent studies in human adults have shown that they are sensitive to crossmodal spatiotemporal dynamics between external objects and the body. In fact, a number of authors now argue that the special status of representations of peripersonal spatial events in the brain and behaviour (shown, e.g., in speeded responses to stimuli close to the body) may be explained by the predictive mechanisms at play when somatosensory processing is modulated by prior visual, auditory or audiovisual stimuli that move towards the body^1,11,27^. Evidence for this account comes from a number of studies demonstrating that responses to tactile stimuli can be modulated by predictive but spatially or temporally distant stimuli in a different sensory modality (i.e. vision)^9,10^. The key novelty of these findings is their focus on crossmodal interactions via predictive relations between visual and somatosensory events, which cannot be mediated via exogenous crossmodal effects due to colocation or synchrony between the visual and tactile stimuli^28,29^. These findings support the existence of predictive mechanisms using visual motion cues to make judgments about the time and location of an impending tactile stimulus and enhancing tactile processing at the time and location of impending contact^10^.

A precursory requirement for making predictions about tactile events from preceding visual cues is a sensitivity to visual-tactile crossmodal interactions across spatiotemporal gaps. To investigate the developmental origins of this ability, we presented infants with tactile stimuli on their hands and examined how prior moving visual stimuli modulated subsequent somatosensory evoked potentials (SEPs) recorded from the scalp using electroencephalography (EEG). More specifically, we examined whether somatosensory evoked potentials recorded from the scalp were modulated by the direction of motion of an earlier presented unattended visual object (towards vs away from the body). We decided to convey motion information using visual stimuli because, to sighted humans, these provide the richest and most continuous dynamic spatiotemporal information about objects’ movements with respect to the self, making visuotactile interactions the most important in supporting the development of the ability to represent the relation between objects in external space and the body. Additionally, the choice of visuotactile stimuli is consistent with previous adult research^9,10^. We chose to include in the study two groups of infants aged 4 and 8 months: these age groups were chosen in light of the relevant developmental changes taking place between 4 and 8 months of life. Infants’ ability to localise touch in relation to external spatial coordinates develops between 4 and 6 months^30^ and, relatedly, postural information begins to influence the neural correlates of infants’ tactile perception after 6.5 months of life^31^. Additionally, infants’ ability to reach for and handle objects becomes established from around 5 months^32^. Given the intrinsic link between acting on the environment and perceiving body-related motion in it, we anticipated that the acquisition of sensorimotor experience between 4 and 8 months of age would lead to developmental changes in infants’ visual-tactile processing.

Given the high adaptive value of perceiving and predicting the contact of visual objects with the body, it would be reasonable to expect that the mechanisms supporting it develop early in life^12^. At the same time, multisensory postnatal experience likely plays an important role in shaping the ways in which infants come to integrate visual stimuli specifying imminent contact and subsequent tactile stimuli taking place at the expected time and location of contact. This is particularly relevant with regards to visual moving stimuli, which, unlike auditory ones, can only be experienced by infants in their postnatal life. For these reasons, and in light of the aforementioned important developmental changes taking place during the first year of life^30–32^, we expected to find evidence of developmental changes in the visual modulation of touch in both of the age groups involved in this study. Predicted perceptual outcomes are known to enhance early perceptual components in event-related potentials (ERPs) gathered from 1-year-old infants^33^. Based on this, we expected that any modulation of SEPs observed in either age group would be in the form of an enhanced response to the tactile stimulus following approaching motion. This hypothesised pattern of results would also be consistent with the finding that when adults anticipate a tickling sensation they show enhanced activity in contralateral primary somatosensory cortex^34^.

In this study, we presented infants with tactile stimuli on their hands preceded by dynamic visual stimuli on a screen, rendered to specify 3D trajectories either approaching their hands or moving away from them. The dynamic visual stimuli were presented in the bottom half of the screen, while an attention-getting visual stimulus was presented at the top of the screen. When triggering the presentation of the experimental stimuli, we ensured that the infants were focusing on the attention getter. The approaching display showed a small ball approaching the participants’ hands and disappearing halfway through its trajectory from its starting point to the infant’s hands, while the receding display was the approaching sequence played backwards. After an interval, ensuring that there was no spatial nor temporal proximity between the visual and the tactile stimuli, the infants felt, on 50% of the trials, a tactile stimulus on both hands, which were held at the expected location of contact as signalled by the approaching visual motion trajectory. We recorded the infants’ brain activity throughout the study using EEG and analysed the SEPs measured in response to the tactile stimuli. We calculated a difference waveform between the trials with and without the tactile stimulation to measure and compare purely the somatosensory responses and ensure that any event related electrical activity on the scalp that was driven purely by the visual elements of the stimulation, common across all trials, was removed by the subtraction. We then compared the difference waveforms for the approaching vs the receding visual motion conditions, to explore the influence of unattended visual motion direction on the processing of a subsequent tactile stimulus on the body.

## Results

To compare the responses to the tactile stimulus in the approaching vs receding visual motion conditions we used two complementary analysis strategies. Firstly, we ran a sample-point by sample-point simulation, which allowed us to analyse the full time-course of the response controlling for the correlation between consecutive sample points. The reason for running this analysis, which is common to other studies investigating infant SEPs^31,35^, lies in the limited number of reference studies available to define a-priori the latency of the components of interest. Secondly, we looked more specifically at each of the prominent features identifiable in the grand averaged waveforms by comparing the mean individual amplitude of the response between conditions. This analysis allowed us to further reconfirm the findings of the simulation and at the same time to complement them, given that sample-point simulations are insensitive to waveform differences occurring in brief periods during the ERP epoch.

Because SEP components change significantly in amplitude and latency during the first year of life^36,37^, we treated the responses of the 4- and the 8-month-old infants separately.

### 4-month-olds

**Figure 1:**
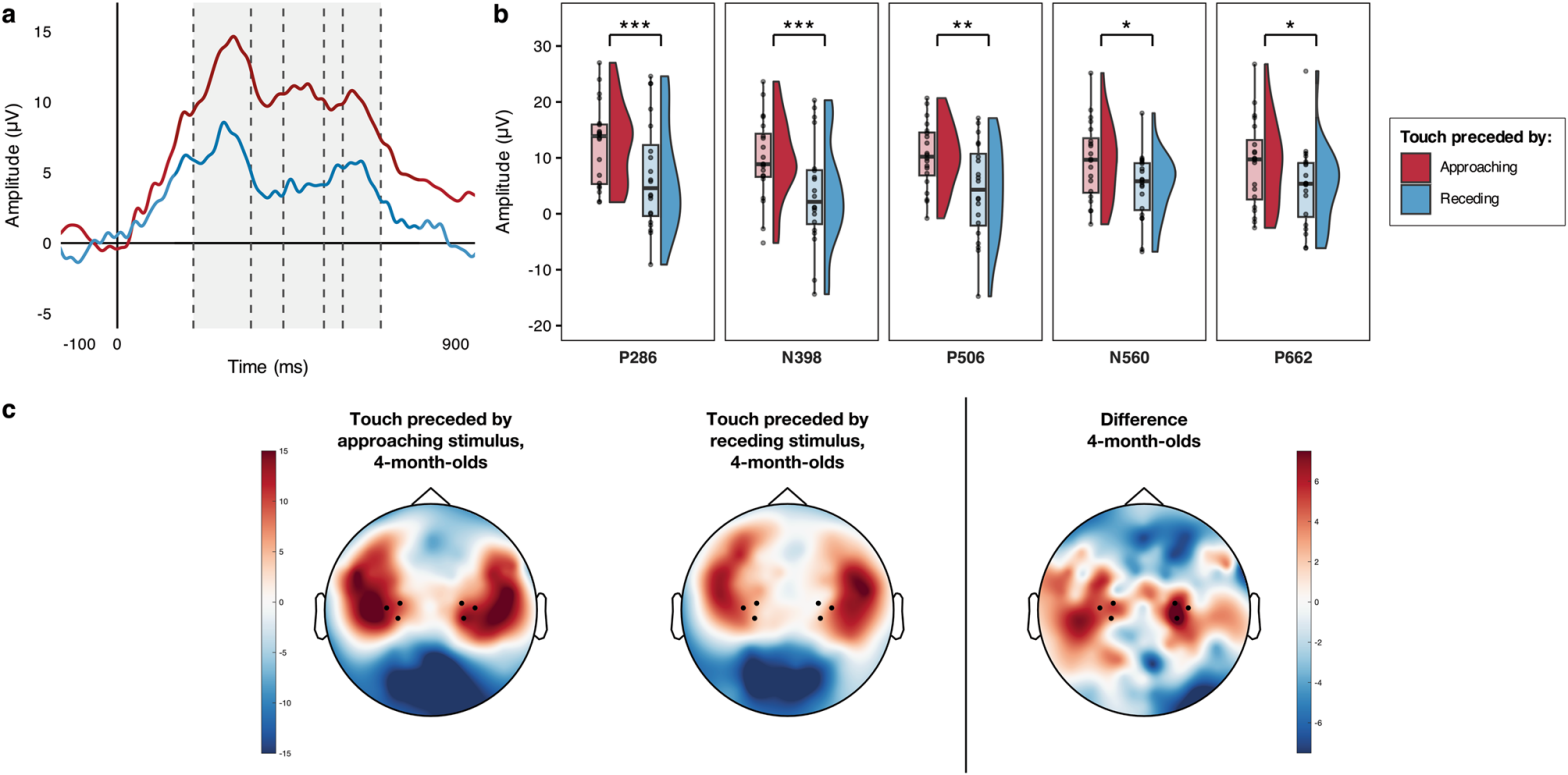
Modulation of 4-month-old infants’ SEPs by the direction (approaching vs receding) of prior unattended visual motion. (**a**) Grand average SEPs across hemispheres; the statistically reliable difference identified by the sample-point by sample-point analysis is indicated by the light grey shading; dashed lines separate the components that were subjected to statistical comparison. (**b**) Voltage differences in the grand averaged mean individual amplitude of the SEPs in the two conditions for the five components of interest; significant comparisons are indicated (*p < 0.05, **p < 0.01, ***p ≤ 0.001). (**c**) Grand average topographical representations of the voltage distribution over the scalp in the two conditions between 202 and 700 ms following the tactile stimulus onset (this was the time-window during which the sample-point by sample-point analysis revealed a statistically reliable difference), with a Touch following Approaching motion—Touch following Receding motion difference map to the right; channels averaged for the analyses are highlighted (36, 41, 42 in the left hemisphere and 93, 103, 104 in the right hemisphere).

First, we ran simulations to determine whether any differences between conditions in the 4-month-olds SEPs were statistically reliable on a sample-point by sample-point basis. This simulation approach examined differences in the SEPs at each sample point between 100 ms prior to the tactile stimulus onset and 900 ms after it, correcting for the auto-correlation of consecutive sample points^38^. The simulation generated 1000 random datasets with the same level of auto-correlation of the observed data, as well as the same number of participants and sample points. Two-tailed one sample t-tests comparing the SEP difference waves (by Condition) vs. zero (α = 0.05, uncorrected) were applied to the simulated data at each time point. In each of the 1000 simulations the longest sequence of consecutive significant t-test outcomes was computed. We then used the 95th percentile of that simulated distribution of “longest sequence lengths” to determine where a sequence of differences in the SEP by Condition were statistically reliable. Here the simulation identified as statistically reliable any sequences of consecutive significant t-tests longer than 220 ms, and thus highlighted a statistically reliable sequence between 202 and 700 ms after the onset of the tactile stimulus (Fig. 1a). This showed, in agreement with our hypotheses, a larger SEP in response to the tactile stimuli following approaching vs. receding motion.

Next, we examined each of the salient components (i.e., prominent features) in the SEPs, comparing their amplitudes across conditions. As we had no prior information to determine the latency of the components of interest, we isolated the main components identifiable within the time window of significance^35^ using a collapsed localisers approach^39^, which reduces the chance of biased measurements by defining the latency of the components to be analysed on the average waveform across conditions. We identified five main components, labelled according to their polarity and peak latency: P286 (202–354 ms); N398 (356–440 ms); P506 (442–548 ms); N560 (550–598 ms); P662 (600–700 ms). Within each component, we ran a paired t-test to compare the mean individual amplitudes of the waveform between the two conditions (Fig. 1b). The comparisons confirmed that in all the identified components the mean individual amplitude of the SEPs was significantly larger when the tactile stimulus had been preceded by approaching motion compared to when it had been preceded by receding motion (Table 1).

**Table 1:** Planned paired comparisons of the modulation of salient components by the direction (approaching vs receding) of prior unattended visual motion in the 4-month-olds group.

Component	*D* value	*p* value	dfs	*t* value	*p* value	*d*_*z*_
P286 (N = 20)	0.111	0.945	19	3.717	0.001	0.831
N398 (N = 20)	0.165	0.591	19	3.803	0.001	0.850
P446 (N = 20)	0.119	0.909	19	3.463	0.003	0.774
N560 (N = 20)	0.157	0.652	19	2.518	0.021	0.563
P622 (N = 20)	0.115	0.928	19	2.490	0.022	0.557

The table summarises the results of the comparisons (two-tailed) of the mean individual average of the SEPs amplitudes in the Approaching vs Receding conditions for each salient component occurring between 202 and 700 ms following stimulus onset; α = 0.05 for all comparisons. Kolmogorov–Smirnov *D* statistics identifying significant deviations from normality in the distributions of the differences between conditions are also included.

### 8-month-olds

**Figure 2:**
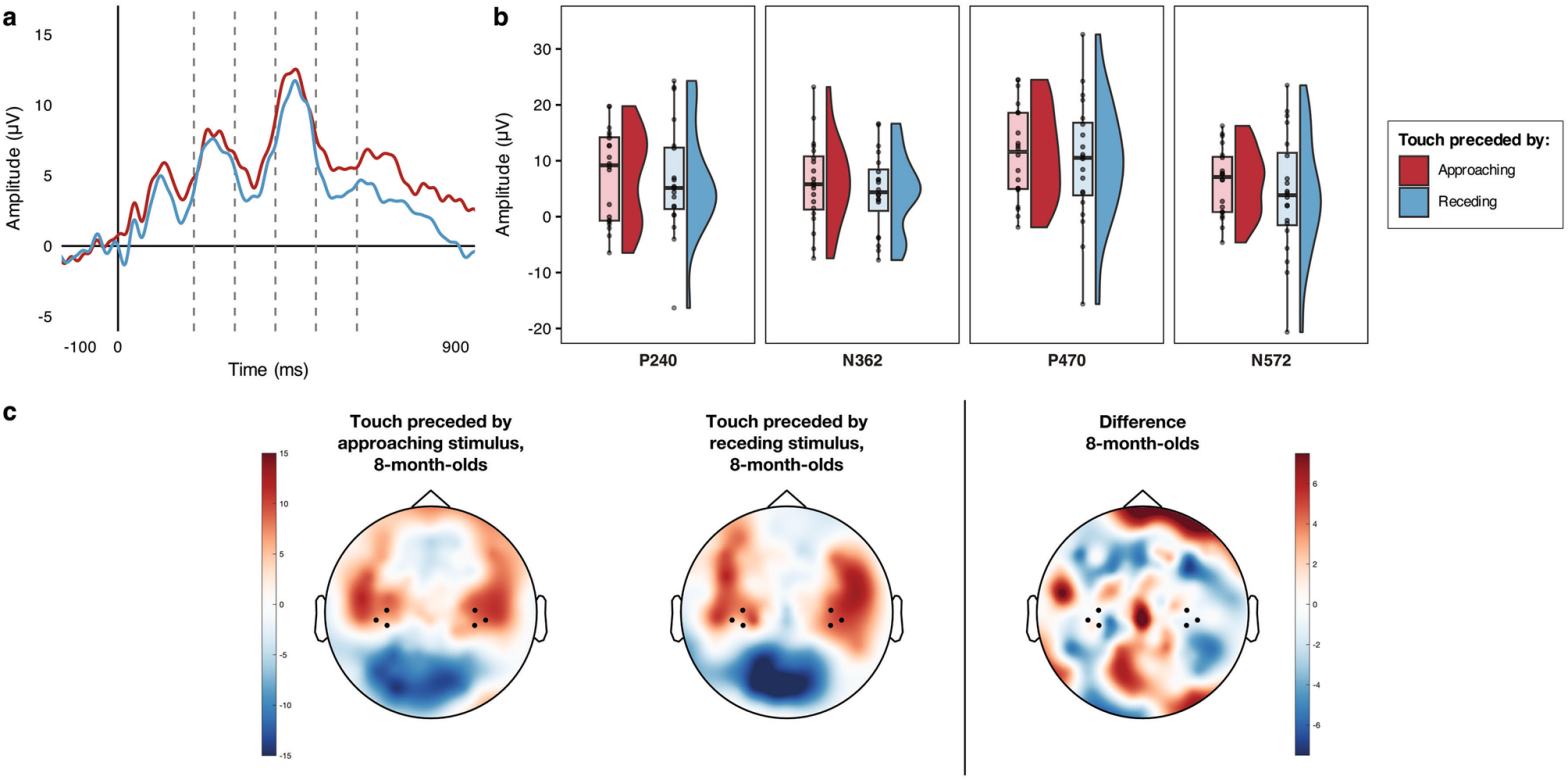
Modulation of 8-month-old infants’ SEPs by the direction (approaching cs receding) of prior unattended visual motion. (**a**) Grand average SEPs across hemispheres; dashed lines separate the components that were subjected to statistical comparison. (**b**) Voltage differences in the grand averaged mean individual amplitude of the SEPs in the two conditions for the four components of interest. (**c**) Grand average topographical representations of the voltage distribution over the scalp in the two conditions between 202 and 700 ms following tactile stimulus onset (this was the time-window during which the sample-point by sample-point analysis revealed a statistically reliable difference in the 4-month-olds group), with a Touch following Approaching motion—Touch following Receding motion difference map to the right; channels averaged for the analyses are highlighted (41, 46 47 in the left hemisphere and 98, 102, 103 in the right hemisphere).

As for the younger age group, we first ran simulations to determine whether any differences between conditions were statistically reliable on a sample-point by sample-point basis. The simulation identified as statistically reliable any sequences of consecutive significant t-tests longer than 106 ms. Based on this criterion, no sequences of statistically reliable differences between conditions were found in the 8-month-olds SEPs (Fig. 2a). It is important to note that this method is insensitive to waveform differences occurring on brief segments of time^38^, and so, as in our analysis of the 4-month-olds SEPs, we further probed those components (i.e., prominent features) that were identifiable within the time window of differences highlighted in the 4-month-olds group (i.e., between 202 and 700 ms after the tactile stimulus onset). Four components were identified, and labelled according to their polarity and peak latency: P240 (202–310 ms); N362 (312–418 ms); P470 (420–526 ms); N572 (528–636 ms). For each component, t-tests comparing the mean individual amplitude of the response between conditions revealed no significant differences, confirming the findings from the simulations reported above (Table 2 and Fig. 2b).

**Table 2:** Planned paired comparisons of the modulation of salient components by the direction (approaching vs receding) of prior unattended visual motion in the 8-month-olds group.

Component	*D* value	*p* value	dfs	*t* value	*p* value	*d*_*z*_
P240 (N = 20)	0.194	0.387	19	0.490	0.630	0.109
N362 (N = 20)	0.133	0.828	19	0.867	0.397	0.194
P470 (N = 20)	0.118	0.913	19	0.599	0.556	0.134
N572 (N = 20)	0.122	0.894	19	0.937	0.361	0.210

The table summarises the results of the comparisons (two-tailed) of the mean individual average of the SEPs amplitudes in the Approaching vs Receding conditions for each salient component occurring between 202 and 700 ms following stimulus onset; *α* = 0.05 for all comparisons. Kolmogorov–Smirnov *D* statistics identifying significant deviations from normality in the distributions of the differences between conditions are also included.

The absence of any effects of the direction of prior and unattended visual motion on somatosensory processing in 8-month-olds is surprising considering the robust effect of Condition observed in the 4-month-olds. To better understand this developmental change between 4 and 8 months of age, we conducted further exploratory analyses on the 8-month-olds’ data, investigating whether any differences between conditions could be masked by individual variations in the participants’ developmental status. Using age in days as a proxy for developmental status, we fitted 3 linear mixed-effects models (LMMs)^40^ for each of the four main components identified (P240, N362, P470, N572), including a categorical fixed effect (Condition), a continuous fixed effect (Age in days) and a random effect (the individual Participant). The first model (*m1*) included only Condition as a fixed effect and Participant as a random effect; the second (*m2*) added Age as a fixed effect; the third (*m3*) added the Interaction between Condition and Age. The models were fitted using the R software^41^, specifically the “lme4” and “lmerTest” packages for LMMs^42,43^.

**Figure 3:**
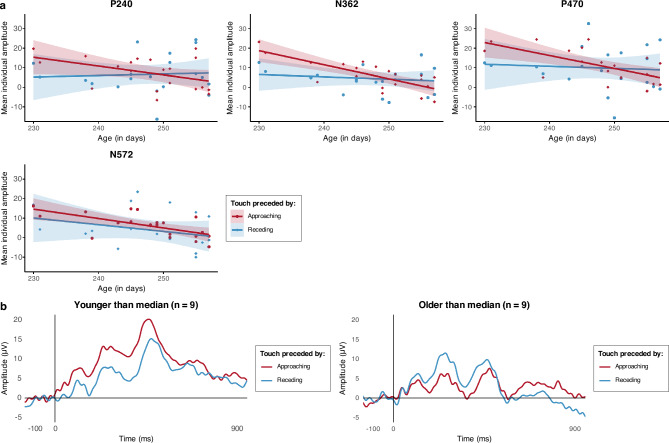
Effect of condition and age (in days) on the SEPs of 8-month-old infants. (**a**) Scatter plots illustrating, for each component of interest, the relation between the mean individual amplitude of the SEPs in each condition and the infants’ age in days, with regression lines (and S.E.M., shaded) for each condition. (**b**) For illustrative purposes, in light of the results of the LMMs, we plotted the grand averaged SEPs for the younger and the older 8-month-olds (2 groups of 9 infants each, created based on the median age value, 249 days); the plots suggest that the direction of the difference between the SEPs in response to a tactile stimulus following approaching vs receding motion might reverse between the younger and the older 8-month-old infants.

Likelihood Ratio Tests (LRTs) were conducted to compare how well the three models explained the data (Table 3). In the first 3 components (P240, N362 and P470), *m3* explained the collected data better than any other model. This model included the interaction between Condition and Age, which was significant across all three components [P240: *t*(18) = 2.662, *p* = 0.016; N362: *t*(18) = 2.916, *p* = 0.009; P470: *t*(18) = 2.135, *p* = 0.047]. This showed how, with increasing age in days, infants SEPs changed from an enhanced response to tactile stimuli preceded by approaching visual motion to an enhanced response to tactile stimuli preceded by receding visual motion (Fig. 3a). Additionally, the models highlighted a significant main effect of Condition once Age and the random effect of Participants were taken into account [P240: *t*(18) = − 2.679, *p* = 0.015; N362: *t*(18) = − 2.947, *p* = 0.009; P470: *t*(18) = − 2.155, *p* = 0.045]. In the fourth component (N572), *m2*, including the fixed effects of Condition and Age, and the random effect of Participant, was the best fit. Reflecting the better fit compared to *m1* there was a main effect of Age [*t*(18) = − 2.215, *p* = 0.04]: this describes, across both conditions, a decline in the SEPs amplitude with age (Fig. 3a).

**Table 3:** Likelihood Ratio Tests comparisons for the three LMMs (m1, m2, and m3) carried out on each observed SEP component.

Component	*m2* vs *m1*			*m3* vs *m1*			*m3* vs *m2*		
	df	*χ﻿*^2^	*p* value	df	*χ*^*2*^	*p* value	df	χ﻿ 2	*p *value
P240	1	0.677	0.411	2	7.317	0.026*			
N362	1	7.598	0.006**				1	7.739	0.005**
P470	1	2.876	0.090	2	7.390	0.025*			
N572	1	4.822	0.028*				1	0.246	0.620

The table summarises the results of the LRTs comparing the 3 models used to analyse the effects of Condition, Age (in days) and their interaction on the SEPs of 8-month-old infants; significant comparisons are indicated (*p < 0.05, **p < 0.01).

Altogether, these results indicate that the amplitude of 8-month-olds’ SEPs were modulated by whether, prior to the tactile stimulus, they were presented with an unattended visual object approaching them or receding away from them. This was demonstrated via an interaction between the direction of the visual motion and age in days in affecting the amplitude of SEP components. More specifically, the younger 8-month-old participants showed, as did 4-month-olds, a larger response to tactile stimuli preceded by approaching motion, while the older 8-month-olds participants showed the opposite pattern (Fig. 3b).

For completeness, we fitted the same models for the 4-month-old infants. In this group, neither *m2* nor *m3* significantly improved on the fit of *m1*, which included only Condition as a fixed effect and Participant as a random effect (Supplementary Table S1). This result, obtained for all components tested, confirms our previous findings that 4-month-old infants demonstrate an enhancement of the response to the tactile stimulus when it was preceded by unattended approaching vs receding visual motion, irrespective of the participants’ age in days (Supplementary Fig. S1).

## Discussion

From at least 4 months of age human infants’ cortical somatosensory processing is modulated by the direction of movement relative to the body of a previously presented (and unattended) visual object. Previous studies have already identified that young infants are sensitive to the movement of visual objects with respect to the body^19,20,24^, and to spatial and temporal correspondences between tactile and visual stimuli on their body^21–23^. However, the current study shows for the first time that young infants can combine these abilities and are thereby sensitive to the dynamic relations between visual and tactile stimuli when these stimuli are presented in different places (in the external space and on the body) and across a temporal gap (in this case 333 ms between the disappearance of a moving visual object on a screen, and the onset of a somatosensory stimulus on the hands). This is important because such an ability is a prerequisite for infants to be able to perceive the multisensory interactions that characterise the dynamic interactions between objects and the body in peripersonal space.

Our findings show that 4-month-olds’ (and younger 8-month-olds’) SEPs are enhanced following approaching visual motion, while older 8-month-olds seem to show the reverse pattern, with larger SEPs in response to tactile stimuli following receding motion. Overall, it is clear that in both 4- and 8-month-old infants visual information specifying motion in relation to the observer (towards or away from their body), even when not fixated upon visually, affects subsequent somatosensory processing. The present findings therefore prompt the striking conclusion that human infants, from as young as 4 months of age, can coordinate multisensory information presented across different locations (the body and the external space) and at different times (i.e., when one stimulus is predictive of the following one). This fundamental multisensory ability is among those that enable mature humans and animals to perceive their bodily selves in their dynamic relationships with the world around them.

Having established that this sensitivity to tactile-visual interactions in peripersonal space is available at 4 months of age is significant. Infants do not typically make successful reaches to objects that they have targeted in vision before 5 months of age^32^, and so the current findings indicate that infants can learn about the visual-tactile structure of peripersonal space despite an extremely limited ability to undertake skilled action in the environment. This is not to say that pre-reaching infants are not exposed to rich visual-tactile multisensory experiences in the first postnatal months. Indeed, we might speculate that the associations between visual motion and tactile contact that are necessary to explain the present findings might in part result from multisensory input arising from rich interpersonal infant-carer interactions occurring during activities such as feeding and tickling. Further research investigating the nature of early multisensory experiences in the first months of life^44^ could shed valuable light on the early origins of infants’ perceptions of the links between their bodies and the visual environment.

Studies of the neural basis of peripersonal space in mature primates^5,45^ and human adults^46–48^, have identified a neural network subserving fast neural and behavioural responses to objects and sounds approaching the body. These responses are thought to be underpinned by predictive mechanisms using visual motion cues to make judgments about the time and location of an impending tactile stimulus and subsequently enhancing tactile processing at the time and location of impending contact^10^. Our findings show that from 4 months of age human infants are capable of representing dynamic crossmodal spatiotemporal links between visual stimuli and subsequent tactile events. Although it seems reasonable that this multisensory perceptual capacity would be a necessary developmental precursor of an ability to make predictions about tactile contact on the basis of prior visual events, based on our current findings we cannot conclude that infants of 4 and 8 months of age are able to make such predictions.

One possible account of our findings that does not rest on a predictive ability is that the multisensory interactions reported here are mediated by crossmodal spatial attention^49^. For instance, in the Approaching condition, the visual object moving towards the infant may have cued them to (covertly) shift their attention towards the body where the tactile stimulus was later presented (NB: any trials where an overt eye movement was made were excluded). To our knowledge, no research has yet investigated crossmodal effects in selective attention in human infancy, therefore at present there is no directly relevant evidence to determine whether this is a plausible account of our findings in 4- and 8-month-old infants. Nonetheless there is evidence that both exogeneous and endogenous orienting mechanisms are available within the same sensory modality by 4 months of age^50^, though with considerable development of endogenous orienting through to childhood and beyond^51–53^. Furthermore, given the evidence of the development of endogenous attention in the first year of life^52^ we might consider whether this could contribute to explain the rapid changes in the SEPs that we observed in 8-month-old infants in the present study.

Another possible explanation of the reported modulation of young infants’ SEPs by visual stimuli may be that the SEPs reflect motor preparation responses triggered by the visual cues^54^. Indeed, as Eimer et al.^54^ show, in adults the mechanisms involved in attentional shifts and motor preparation appear to be very similar. Additionally, it has been demonstrated that approaching visual stimuli can trigger, in adults, the activation of sensorimotor networks responsible for the preparation of a timed motor response^55,56^. Nonetheless, it is of note that we have found evidence of a modulation of the SEP by prior visual events in infants as young as 4 months of age. At this age infants would generally not have obtained the motor skills to coordinate visual and tactile space with the hands or feet^57^ and, as such, it does not seem likely that they would be able to make the rapid motor responses that would be required if the reported effects on their SEPs were entirely driven by motor preparation.

A final possibility is that, even though the visual and tactile stimuli were presented separately in time and space, the infants may have perceived them simultaneously by virtue of an extended visual-tactile temporal binding window. Previous research has demonstrated a developmental narrowing of the visual-tactile temporal binding window in childhood^58^, and it seems reasonable to speculate that there might be an additional narrowing of visual-tactile binding between early infancy and later development. Therefore, there is a possibility that infants’ visual-tactile window might be longer than the temporal interval between the end of the visual stimulation and the beginning of the tactile stimulation. By extension, it may be that despite the spatial and temporal gap between the stimuli, the infants might have showed visual modulations of somatosensory processing because they perceived the visual and tactile events simultaneously. If this is the case, 4-month-old infants’ enhanced SEPs following approaching motion could have resulted from the congruency between approaching motion and a subsequent tactile contact (where this congruency may have been learned from prior encounters with objects approaching the body and touching it).

We have offered four potential accounts of the visual-tactile abilities here observed in 4- and 8-month-olds: that infants are able to process visual-tactile peripersonal events via (i) visual-tactile predictive process, (ii) visual-tactile crossmodal attention, (iii) visual cuing of motor preparation, and (iv) visual-tactile crossmodal binding. None of these accounts are mutually exclusive of the others, and indeed the various perceptual abilities implied may all play roles in supporting infants’ perceptual awareness of events in peripersonal space at various points in development and may be developmentally related to each other. For example, one possibility is that more crude coordination of visual and somatosensory interactions (via an extended visual-tactile temporal binding window), may provide a developmental bootstrap for more complex perceptual representations and skills used by older infants, children, and adults (e.g., visual-tactile crossmodal spatial attention and/or rapid visual-tactile predictive processing). In order to be able to differentiate between these possibilities and their developmental relationships, researchers will need to employ both neural and behavioural measures of multisensory functioning within a number of cross-sectional and longitudinal designs. Crucially however, all these accounts imply that from 4 months of age, infants have a means to coordinate their somatosensory (bodily) responses in a temporally contiguous way with prior dynamic visual information presented in extrapersonal space, thereby supporting a means of representing multisensory peripersonal events.

Lastly, we consider the differences between the 4- and 8-month-olds’ processing of visual tactile peripersonal events reported here. We observed that the enhancement of somatosensory responses following visual approach seen in 4-month-olds was not apparent in 8-month-olds. We also reported some exploratory analyses that tentatively established a relation between 8-month-old infants’ age in days and the modulation of their SEPs in response to somatosensory stimuli preceded by visual approaching or receding motion. The enhancement seen in the 4-month-old infants was also seen in the younger 8-month-olds, but gradually reversed with age in days such that by 260 days of age, infants show enhanced SEPs following receding motion on prominent components (P240, N362 and P470). These observed differences between 4- and 8-month-olds’ SEPs could indicate developmental changes in the brain and behavioural basis of peripersonal spatial representations, and this possibility is commensurate with a number of findings from other studies concerning the development of body representations and somatosensory processing in early life^30,31,59^. An alternative developmental explanation in the current study is attentional control. It may be that the older 8-month-old infants were able to better focus on the attention getter and to ignore the peripheral visual motion leading to smaller enhancements of their SEPs. Nonetheless, we consider this alternative explanation unlikely as, even in adults whose attentional control is more efficient than infants’, the detection of tactile stimuli is facilitated by unattended approaching visual motion^9,10^. Another alternative account is that the developmental trend towards increasing enhancement of SEPs in the receding condition represents the development of a neural process involved in signalling prediction error (i.e., that the tactile contact experienced following receding motion is unexpected). As such, the gradual emergence across the 8-month-old group of greater responses to unexpected tactile contact might represent the emergence of top-down influences of predictions on the perceptual processing of somatosensory information^60^. Based on this finding, we may speculate that if a group of adults were presented with these same stimuli, they would show a pattern of SEP responses like that shown by the older 8-month-old participants reported here. Research into infants’ sensory predictions^33,61–64^, has shown that, even in the first year of life, infants’ early sensory processing can be modulated by top-down influences, suggesting that infants can generate an internal model of the environment and form predictions about it^63,65^. The age-related changes in the visual modulation of SEPs reported here may represent a feature of the emergence of an ability to predict tactile outcomes on the body based on the prior movements of visual stimuli in extrapersonal and peripersonal space.

Here we have shown that, from as early as 4 months of age, infants process somatosensory information differently when it has been preceded by a temporally and spatially distant visual object approaching the body. This finding indicates that fundamental aspects of the multisensory processes underpinning peripersonal space representations, and self-awareness more generally, are in place prior to the onset of skilled action. Nevertheless, there are striking developmental changes in how infants’ brains process visual-tactile events occurring across peripersonal space between 4 and 8 months of age. As infants approach 9 months we increasingly see, in later somatosensory components, a greater processing of those tactile stimuli that were not predicted by preceding unattended visual motion. These findings yield exciting new clues to the ontogeny of human self-awareness in the first year of life, suggesting important postnatal developments in the ability to form expectations about the interactions between the body and the external environment.

## Method

### Participants

The 4-month-old age-group included 20 infants (9 female) aged on average 125 days (4.09 months, *SD* = 9.24 days, range between 109 and 137 days, recruited between 3.5 and 4.5 months of life). Further 31 4-month-olds participated, but were excluded due to restlessness or lack of interest in the screen (n = 9), insufficient artefact-free trials (n = 5), high impedance around the reference (n = 1), or experimental error (n = 16). The 8-month-old age-group included 20 infants (10 female), aged on average 248 days (8.11 months, *SD* = 8.06 days, range between 230 and 257 days, recruited between 7.5 and 8.5 months of life). Further 34 8-month-olds participated, but were excluded due to restlessness or lack of interest in the screen (n = 14), insufficient artefact-free trials (n = 3), high impedance around the reference (n = 3), or experimental error (n = 14). This rejection rate is not unusual for ERP research in infancy^66^, particularly in studies, like the current, where infants are required to attend each stimulus for a number of seconds^67^. As no previous studies used similar designs and manipulations, there were no strong expectations concerning effect sizes. The sample size for each age group (n = 20) was therefore determined based on prior studies using a similar method with infants of similar age^35,68^. The selected sample size was later corroborated by a power analysis based on a study investigating the modulation of SEPs in 6.5- and 10-month-old infants^31^. This study indicated an effect size of *d* = 0.73 for the effect of condition on the SEPs, which given *α* = 0.05 and with an expected power of 0.85 would require a sample of 19 infants.

Testing took place when the infants were awake and alert. The parents were informed about the procedure and provided informed consent for their child’s participation. The participants were recruited from the Goldsmiths InfantLab database and received a small gift to thank them for their participation. Ethical approval was gained from the Institutional Ethics Committee, Goldsmiths, University of London, and the study was conducted in accordance with the Declaration of Helsinki.

### Design

The study included two conditions, based on the direction of the visual motion events presented: Approaching and Receding. Each condition comprised two types of trials: Touch and No-Touch. In both types of trials of each condition, the infants were presented with a set of visual events on screen, including an attention-getter in the top half of the screen and visual motion events in the bottom half. In the Touch trials, they were also presented with vibrotactile stimuli on both hands. The No-Touch trials were included to ensure that the visual elements of the stimulation could be subtracted from the somatosensory responses (see “EEG recording and analyses”).

The trials were presented in groups of 4, within which each condition and trial type were presented in a random order, and grouped in blocks of 8 trials each. After each block, a 12 s video was presented to break up the repetitiveness of the stimuli. Each infant was presented with blocks of trials until their attention lasted, up to a maximum of 10 blocks.

### Procedure, stimuli and apparatus

Each infant sat on their parent’s lap on a chair in front of a 24″ screen in a dimly lit room. The parent kept their child’s hands close together about 30 cm away from the screen and held them as still as possible, by holding the wrists. While this led to tactile stimuli for the infant, such touches are very unlikely to have had an impact on the reported SEPs for two reasons: (i) the tactile stimulation from the parents was a varying constant throughout the study, and not time-locked to the experimentally presented tactile stimuli, and was therefore unlikely to have an impact on event-related responses to time locked tactile stimuli; (ii) any changes in the parents’ holding of their infants’ wrists (e.g., repositioning, changes in pressure), would be completely random across participants and conditions, and therefore very unlikely to influence grand averaged ERPs or differences between conditions. An infant-friendly video attracted the infant’s attention to the screen: as soon as the infant was looking at the screen, the experiment began.

Throughout each trial, the infant was shown an “attention-getting” animal character face in the top half of the screen. This was constantly rotating, alternately clockwise and anticlockwise (the first motion direction was counterbalanced across participants), between an orientation where the upper portion of its vertical axis was 45° clockwise from the vertical, and one where it was 45° anticlockwise from the vertical. The animal face (22.17° × 20.43°) was randomly selected from a set of 10 on a trial-by-trial basis. The attention-getter was intended to attract and hold the participant’s attention during the trial, in particular when the visual moving stimulus was presented: any trials where the infant looked away from the attention getter during the presentation of the visual moving stimulus were identified by offline coding and excluded from the analyses.

Once the infant had fixated the attention-getter (verified via live video feed), the experimenter triggered the stimuli. A 3D rendered red ball (5.90° × 5.57° at its smallest) appeared in the lower half of the screen and either approached the infant’s hands or receded towards the background, moving for 333 ms. The duration of the motion was based on a previous study with adult participants^10^ and doubled to ensure that young infants could perceive the visual motion. The ball moved within a 3D rendered room, whose width, measured at the bottom of the screen, was 40 cm. This measure was used as a common reference between the real and the simulated worlds to calculate the distance of the simulated background from the screen surface. We wanted the screen surface to be perceived as halfway between the background and the infant’s hands, which were 30 cm away from the screen. The 40 cm width of the simulated room corresponded to 28 measurement units in the rendering software, therefore we located the background 21 measurement units away from the front, at a simulated distance of 30 cm from the screen surface and of 60 cm from the participants’ hands. In the Approaching condition, the ball moved from the background towards the infant’s hands but disappeared when the rendering specified that it reached halfway through its trajectory. In the Receding condition, the ball moved from the halfway point towards the background.

**Figure 4:**
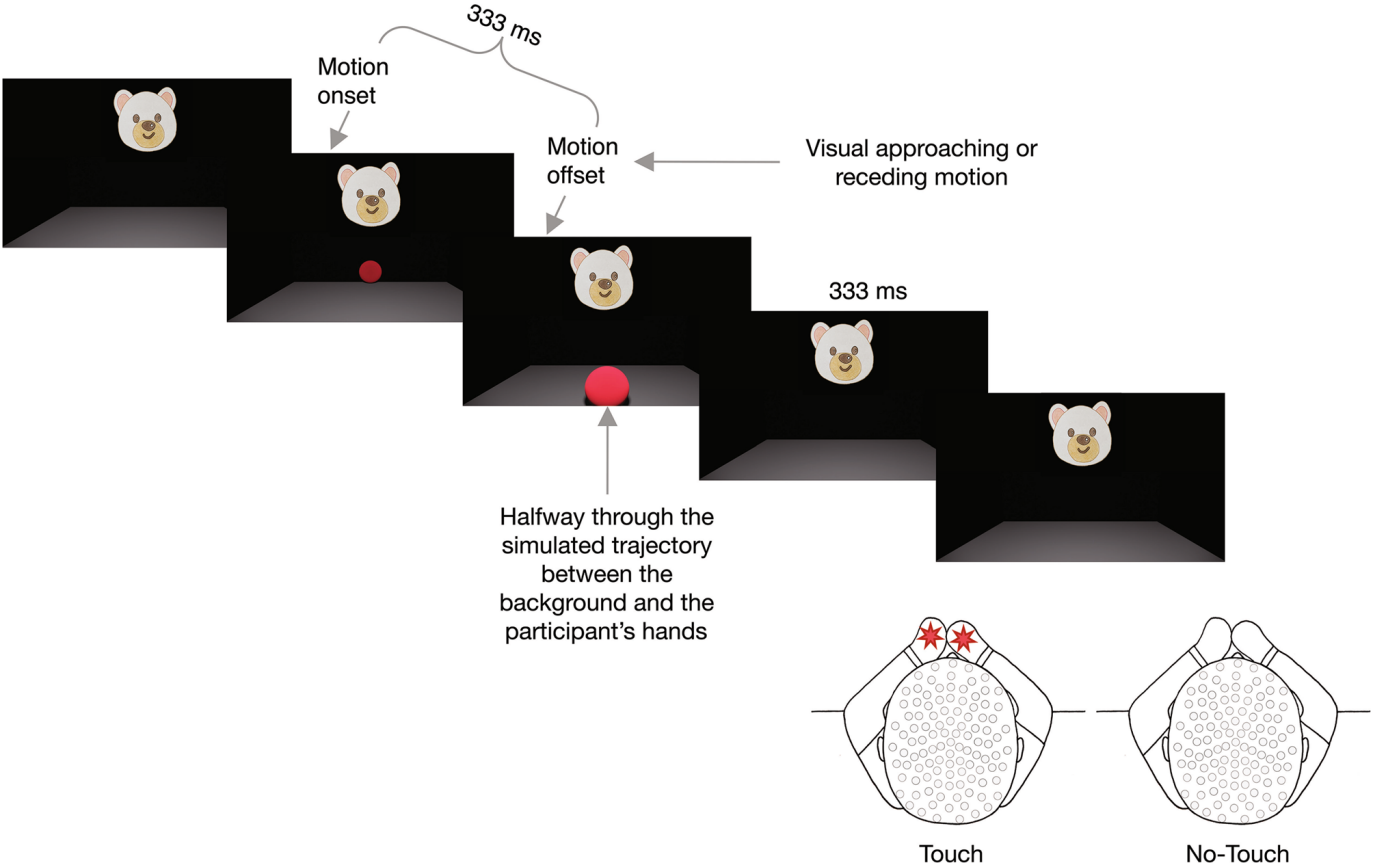
Schematic representation of the experimental procedure. When the infant fixated on the attention-getting animal face, the experimenter triggered the presentation of the experimental stimuli. A red ball appeared in the lower half of the screen and either approached the infant’s hands or receded towards the background for 333 ms. After a 333 ms interval, the infant received, on 50% of the trials, a vibrotactile stimulus on both hands, lasting 200 ms. During the study, the parent was instructed to keep the infant’s hands close to each other and along the midline, i.e., along the simulated trajectory of the moving ball.

In both conditions, after the ball disappeared there was an interval (333 ms) followed, in the Touch trials only (50% of the total), by a 200 ms vibrotactile stimulus on both hands (Fig. 4 and Supplementary Movie S1). The interval before the tactile stimulus lasted as long as the motion: as the ball disappeared when it reached the simulated halfway point between the background and the infant’s hands, this ensured that the tactile stimulus was presented at the expected time to contact of the simulated moving ball with the hands in the Approaching condition.

Each trial lasted minimum 4 s, including: minimum 2 s when only the attention-getter was presented, 333 ms of visual motion, the 333 ms interval, and 1333 ms of response collection time, whose first 200 ms corresponded to the tactile stimulation in the Touch trials. The duration of the response collection time was chosen to ensure that the SEPs in response to each tactile stimulus had completely subsided before a new tactile stimulus was presented.

The vibrotactile stimuli were delivered via custom-built voice coil tactile stimulators (tactors), driven by a 220 Hz sine wave. One tactor was placed in each of the infant’s hands and fixed to the palms with self-adherent bandage; the infant’s hands and the tactors were covered with cotton mittens. An audio track made of a lullaby and white noise was played ambiently to mask the noise of the tactors. The 3D stimuli were rendered using Blender 2.79b (Blender Foundation, Amsterdam, Netherlands); the stimuli were presented using MatLab 2006a (7.2.0.232) and Psychtoolbox 3 3.0.9 (beta).

### EEG recording and analyses

The participants’ electrical brain activity was continuously recorded using a Hydrocel Geodesic Sensor Net (Electrical Geodesics Inc., Eugene, Oregon), consisting of 128 silver-silver chloride electrodes evenly distributed across the scalp and referenced to the vertex. The potential was amplified with 0.1 to 100 Hz band-pass and digitized at 500 Hz sampling rate^67^. The raw data were processed offline using NetStation 4.5.1 (Electrical Geodesics Inc., Eugene, Oregon). Continuous EEG data were high-pass filtered at 0.3 Hz and low-pass filtered at 30 Hz using digital elliptical filtering^67^. They were segmented in epochs from 300 ms before the tactile stimulus onset to 1300 ms after it and baseline-corrected to the average amplitude of the 100 ms preceding the tactile stimulus onset. Epochs containing movement artefacts or more than 12 bad electrodes^67^ were visually detected and rejected. Bad electrodes were interpolated trial-by-trial using spherical interpolation of neighbouring channel values. Artefact free data (4mo, M = 9.85, 8mo, M = 9.63, see Supplementary Table S2; small number of trials are usual in infancy research^67^) were re-referenced to the average potential over the scalp, then individual averages were calculated. Additionally, the video recordings of the session were coded offline to identify and exclude any trials where the participant: (i) was not looking at the screen during the presentation of the visual moving stimulus, (ii) was looking at the visual moving stimulus rather than the attention-getter, (iii) had their hands in an incorrect position. The number of trials excluded for due to these codings was exiguous compared to the number of trials presented (see Supplementary Table S3 for details).

We wanted to compare the responses to the tactile stimuli themselves, ensuring that the analysed waveforms did not include any event related electrical activity on the scalp that was driven purely by the visual elements of the stimulation. To achieve this, for both visual motion conditions, we removed any electrical activity driven by the visual elements of the stimulation, which were common across the two types of trials, by computing the difference waveform obtained subtracting the response recorded in the No-Touch trials from that recorded in the Touch trials (see “Design”).

We were interested in the SEP responses recorded from sites near somatosensory areas. To identify the electrode clusters for the analyses, we averaged the difference waves and inspected the topographic maps representing the scalp distribution of the electrical activity^39^, confirming the presence of hotspots in the regions surrounding CP3 and CP4 in the 10–20 system^69,70^ (Figs. 1c and 2c). Next, we visually inspected the recordings from the electrodes within these areas and isolated, for each hemisphere, the electrode cluster showing the most pronounced SEP components^35^: for the 4-month-old group, 36, 41, 42 (left hemisphere) and 93, 103, 104 (right hemisphere); for the 8-month-old group, 41, 46 47 (left hemisphere) and 98, 102, 103 (right hemisphere).

### Supplementary Information

Supplementary Information.

Supplementary Video 1.

## Data Availability

The present study has not been formally preregistered. The datasets generated and/or analysed during the current study are available in the University of Birmingham eData repository and can be retrieved from 10.25500/edata.bham.00000447. The scripts and datasets used to perform the analyses reported in this manuscript are available online on the Open Science Framework website and can be retrieved from https://osf.io/jg7xf/. A version of this manuscript has been posted as a preprint^71^, made available under a CC-BY-NC-ND 4.0 International license, and can be retrieved from: https://www.biorxiv.org/content/10.1101/2020.09.07.279984v1.
