# Supplementary material for: Visual objects approaching the body modulate subsequent somatosensory processing at 4 months of age
Source: Sci Rep. 2023 Nov 21;13:19300. doi: 10.1038/s41598-023-45897-4 (PMC10663495; doi:10.1038/s41598-023-45897-4)
Supplement: Supplementary file 1 — Supplementary Information. [file 41598_2023_45897_MOESM1_ESM.pdf]

Visual objects approaching the body modulate subsequent somatosensory processing at 4 months of age - Supplementary Information

Giulia Orioli, Irene Parisi, José L. van Velzen and Andrew J. Bremner

Supplementary Figure S1

Effect of condition and age (in days) on the SEPs of 4-month-old infants. Scatter plots illustrating, for each component of interest, the relation between the mean individual amplitude of the SEPs in each condition and the infants' age in days, with regression lines (and S.E.M., shaded) for each condition.

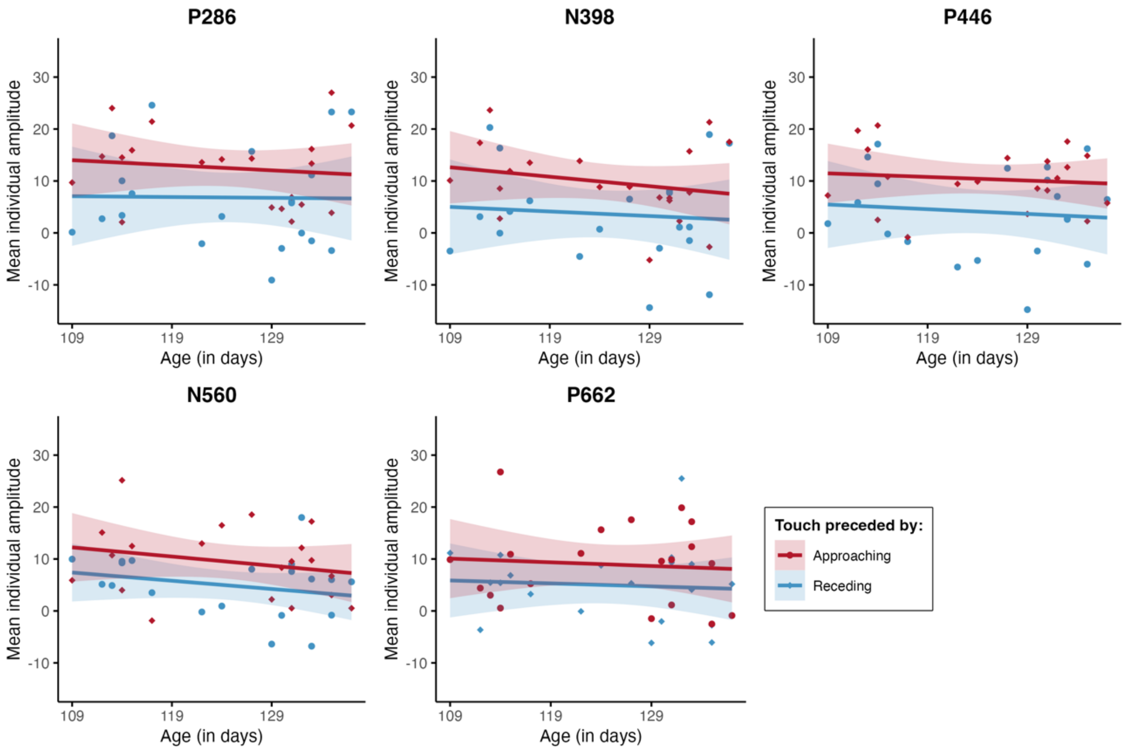

Supplementary Table S1

Likelihood Ratio Tests (LRTs) comparisons for the three LMMs (m1, m2, and m3) carried out on each observed SEP component.

Component	***m2 vs m1***			***m3 vs m1***		
	df	χ^2	*p* value	df	χ^2	*p* value
P286	1	0.085	0.770	2	0.338	0.844
N398	1	0.534	0.465	2	0.825	0.662
P506	1	0.285	0.594	2	0.296	0.862
N560	1	1.949	0.163	2	1.957	0.376
P662	1	0.153	0.696	2	0.160	0.923

Note: The table summarises the results of the LRTs comparing the 3 LMMs used to analyse the effects of Condition, Age (in days) and their interaction on the SEPs of 4-month-old infants.

Supplementary Table S2

Mean number of artefact-free trials for each age group and trial type (parenthetical values are SDs, followed by ranges).

Age Group	Approaching Touch	Approaching No-Touch	Receding Touch	Receding No-Touch
4-month-olds (N = 20)	10.05 (3.15; 7-19)	10.2 (3.72; 7-19)	9.40 (3.12; 7-19)	9.75 (3.6; 7-19)
8-month-olds (N = 20)	9.85 (2.13; 6-16)	9.25 (2.67; 7-18)	9.50 (2.65; 6-15)	9.90 (2.95; 7-18)

Supplementary Table S3

Total number of trials excluded due to behavioural reasons (on the 1st row), for each age group and trial type, with total number of trials presented per age group and condition (on the 2nd row).

Age Group	Approaching Touch		Approaching No-Touch		Receding Touch		Receding No-Touch	
	L.B	H.P.	L.B	H.P.	L.B	H.P.	L.B	H.P.
4-month-olds	2	10	7	5	5	10	2	10
(N = 20)	360		359		359		353	
8-month-olds	15	6	26	4	16	3	21	3
(N = 20)	374		368		374		374	

Note: The columns titled L.B. include the trials excluded due to the infants' looking behaviour (i.e., looking away from the screen during the presentation of the moving stimulus or looking at the moving stimulus itself); the columns titled H.P. include the trials excluded due to the infants' having their hands in a wrong position.

Supplementary Video S1.

Sample of the stimuli from an experimental session. The video shows an 8-month-old participant taking part in the study (the parent of the portrayed infant provided written consent to online open-access publication). In the bottom left corner of the video is shown the experimental stimulus presented to the infant. The appearance of the word "buzz" on the top left corner indicated the delivery of the tactile stimulus to the participant. The sequence is presented twice, the first time at the real speed, and the second time at half speed.
